# Resolving the Two “Bony” Faces of PPAR-γ

**DOI:** 10.1155/PPAR/2006/27489

**Published:** 2006-09-20

**Authors:** Beata Lecka-Czernik, Larry J. Suva

**Affiliations:** ^1^Department of Geriatrics, Reynolds Institute on Aging, University of Arkansas for Medical Sciences, 629 Jack Stephens Drive, Little Rock, AR 72205, USA; ^2^Department of Orthopaedic Surgery, Center for Orthopaedic Research, University of Arkansas for Medical Sciences, Little Rock, AR 72205, USA

## Abstract

Bone loss with aging results from attenuated and unbalanced bone turnover that has been associated with a decreased number of bone forming osteoblasts, an increased number of bone resorbing osteoclasts, and an increased number of adipocytes (fat cells) in the bone marrow. Osteoblasts and adipocytes are derived from marrow mesenchymal stroma/stem cells (MSC). The milieu of intracellular and extracellular signals that controls MSC lineage allocation is diverse. The adipocyte-specific transcription factor peroxisome proliferator-activated receptor-gamma (PPAR-γ) acts as a critical positive regulator of marrow adipocyte formation and as a negative regulator of osteoblast development. *In vivo*, increased PPAR-γ activity leads to bone loss, similar to the bone loss observed with aging, whereas decreased PPAR-γ activity results in increased bone mass. Emerging evidence suggests that the pro-adipocytic and the anti-osteoblastic properties of PPAR-γ are ligand-selective, suggesting the existence of multiple mechanisms by which PPAR-γ controls bone mass and fat mass in bone.

## INTRODUCTION

The two-faced ancient Roman god Janus, represents the inseparable relationship between opposites. The nuclear receptor and transcription factor PPAR-γ has many “faces” in regard to its activities, but its proadipocytic and antiosteoblastic activities in bone closely resemble the two inseparable faces of Janus.

The decreased rate of bone formation and the number of osteoblasts that occurs with aging correlate inversely with an increase in the fat content and a number of adipocytes in the bone marrow [1]. The apparent inverse relationship between osteoblast and adipocyte differentiations and their shared mesenchymal progenitor origin led to the formulation of the hypothesis that binds these two phenotypes and makes them inseparable [2, 3]. According to the shared precursor hypothesis, an increase in adipocyte differentiation occurs at the expense of osteoblast differentiation, and vice versa. However, in some circumstances, adipocytic and osteoblastic differentiation may occur independently [4, 5], suggesting either an existence in adult marrow of separate pools of progenitor cells responding to proosteoblastic and proadipocytic stimuli differently and/or separate regulatory mechanisms of both osteoblast and adipocyte differentiations. This review summarizes the existing evidence supporting either the “simultaneous” scenario or the “independent” scenario. We cite examples, in which the proadipocytic and antiosteoblastic activities of PPAR-γ can be modulated either simultaneously or independently using ligands of different chemical structures. We also summarize the evidence indicating that PPAR-γ is an important regulator of bone homeostasis and marrow mesenchymal stem cell (MSC) differentiation.

**Figure 1:**
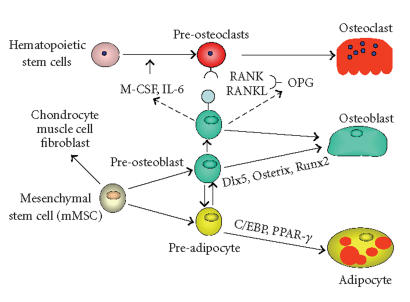
Schematic representation of bone cell development.

Osteoblasts, bone-forming cells, and adipocytes, fat cells, are derived from a common marrow MSC compartment, which also serves as a source of progenitors for fibroblasts, muscle, and cartilage cells, and functions as hematopoiesis-supporting stroma [6, 7]. The commitment of MSCs towards either the adipocyte or osteoblast lineage occurs by a stochastic mechanism [8], in which lineage-specific transcription factors, such as Runx2, Dlx5, and Osterix for osteoblasts and PPAR-γ2 and C/EBPs for adipocytes, are activated (Figure 1) [9].

Aging is associated with changes in the status of MSCs and in the milieu of intrinsic and extrinsic signals that determine the differentiation of MSCs towards osteoblasts and/or adipocytes [1, 10–12]. These changes modulate the continuing dialog between phenotype-specific transcription factors and signals from the microenvironment that collectively determines MSC lineage allocation. With aging, the status of MSCs changes with respect to their differentiation potential, such that commitment to the osteoblast lineage decreases, whereas commitment to the adipocyte lineage increases [1, 10]. These changes in cellular differentiation are reflected in the expression profile of phenotype-specific gene markers in undifferentiated MSCs. The expression of the osteoblast-specific transcription factors, Runx2 and Dlx5, and osteoblast markers, collagen and osteocalcin, is decreased, whereas expression of the adipocyte-specific transcription factor PPAR-γ2 and a gene marker of adipocyte phenotype, fatty acid-binding protein 4 (FABP4), is increased [10]. Aging also results in alterations in the bone marrow microenvironment. MSC support for osteoclastogenesis is enhanced due to the increased production in the marrow of macrophage colony-stimulating factor (M-CSF) and RANKL, two proosteoclastic cytokines required for physiological bone resorption [13–16]. Moreover, bone marrow derived from old mice produces unknown PPAR-γ activator(s) that stimulates adipocyte differentiation and suppresses osteoblast differentiation [10]. Interestingly, in humans osteoblast differentiation can be affected by either a presence of mature marrow adipocytes [17], polyunsaturated fatty acids, which are natural ligands for PPAR-γ [18], or serum derived from older women [12].

## PPAR-γ REGULATES BONE MASS

PPAR-γ nuclear receptor is an essential regulator of lipid, glucose, and insulin metabolism [19]. The receptor is expressed in mice and humans as two different isoforms, PPAR-γ1 and PPAR-γ2, due to alternative promoter usage and alternative splicing [20–22]. PPAR-γ2 differs from PPAR-γ1 by 30 additional amino acids on its N-terminus. PPAR-γ1 is expressed in a variety of cell types, including osteoblasts, whereas PPAR-γ2 expression is restricted to adipocytes, including marrow adipocytes, and is essential for differentiation and maintenance of their phenotype and function [9, 23]. PPAR-γ belongs to the family of nuclear receptor transcription factors, and its activation requires heterodimer formation with another nuclear receptor, retinoid *X* receptor (RXR), and binding of a specific ligand. Natural ligands for PPAR-γ comprise polyunsaturated fatty acids and metabolites of prostaglandin *J*_2_, whereas synthetic ligands include the antidiabetic thiazolidinediones (TZDs) [24].

An important role of PPAR-γ in the maintenance of bone homeostasis has been demonstrated in several animal models of bone accrual [25, 26] or bone loss [27–30], regulated by the status of PPAR-γ activity. Decreased PPAR-γ activity in PPAR-γ-haploinsufficient mice or in mice carrying a hypomorphic mutation in the PPAR-γ gene locus led to increased bone mass, due to increased osteoblastogenesis from bone marrow progenitors, but not due to effects on mature osteoblast activity or cells of the osteoclast lineage [25, 26]. Moreover, age-related osteopenia did not develop in PPAR-γ-haploinsufficient mice [25]. In contrast, activation of PPAR-γ via the administration of rosiglitazone, an antidiabetic TZD, to rodents resulted in significant decreases in bone mineral density (BMD), bone volume, and changes in bone microarchitecture [27–30]. The bone loss observed was associated with the expected reciprocal changes in the structure and function of bone marrow; a decreased number of osteoblasts and an increased number of adipocytes [27, 30]. Indeed, we had previously demonstrated in U-33/γ2 cells, a model of murine marrow mesenchymal cell differentiation, that activation of the PPAR-γ2 isoform by rosiglitazone converted cells of the osteoblast lineage to terminally differentiated adipocytes irreversibly suppressing the osteoblast phenotype via the inhibition of osteoblast-specific gene expression [9].

While the antiosteoblastic effect of PPAR-γ2 on osteoblast differentiation is well established, its effect on osteoclast development is less clear. In vitro, PPAR-γ activation in osteoclast precursor cells inhibits their differentiation [31, 32], whereas activation of PPAR-γ in cells of mesenchymal lineage increases their support to osteoclastogenesis [33]. In vivo, and in contrast to other animal models, bone loss due to rosiglitazone administration to ovariectomized rats resulted from increased bone resorption, but not decreased bone formation [28]. These results indicate that at least in some circumstances, bone loss due to PPAR-γ activation may involve increased bone resorption.

Since TZDs have only been approved for clinical use in the treatment of type II diabetes since 1999, their effects on human bone are just emerging. Early observations indicated that the 4-week administration of troglitazone to patients with poorly controlled type II diabetes who exhibited high bone turnover resulted in a significant decrease in metabolic bone markers, such as urinary deoxypyridinoline, urinary type I collagen C-terminal telopeptide, and serum bone-type alkaline phosphatase [34]. Recent analysis of data from the Health, Aging, and Body Composition cohort indicate that TZD use for more than 3 years results in the acceleration of bone loss, at approximately 1% per year in older postmenopausal women [35].

Emerging evidence from studies of PPAR-γ gene polymorphism in humans strongly suggests a role for this transcription factor in the regulation of bone mass. A silent *C* → *T* transition in exon 6, which is common to both PPAR-γ isoforms, results in a lower bone density and a predisposition to osteoporosis in postmenopausal Japanese women [36]. The same polymorphism in a population of healthy middle-age Korean women was associated with lower levels of circulating osteoprotegerin, a negative regulator of osteoclast development, but no changes in bone density [37]. Another polymorphism in the STAT5B regulatory element in the alternative promoter of the human PPAR-γ1 protein was associated with increased height and plasma low-density lipoprotein cholesterol concentrations in a French population [38]. Similarly, analysis of a population from the Framingham Offspring study revealed several novel polymorphic changes in the coding region of PPAR-γ that correlated independently with bone mineral density (BMD) at different skeletal sites [39]. A more detailed review of the associations between PPAR-γ genomic polymorphism and bone status can be found in this issue [40].

As mentioned above, natural ligands of PPAR-γ include polyunsaturated fatty acids and their oxidized derivatives, the levels of which increase in the circulation with aging. We showed previously that oxidized forms of linoleic acid serve as ligands for PPAR-γ2 in marrow MSC and activate either its proadipocytic and/or antiosteoblastic properties [4]. Oxidized fatty acids are generated in the enzymatic reactions controlled by lipoxygenases. It was demonstrated that three of them, 5-, 12-, and 15-lipoxygenases, are involved in the regulation of bone mass in mice and human. The disruption of either 5- or 15-lipoxygenase in mice led to increased bone mass [41, 42], whereas in humans polymorphic changes in the locus for 12- or 15-lipoxygenases correlated with changes in BMD in normal subjects or in postmenopausal women, respectively [43, 44].

Age-related osteoporosis is typified by a low serum IGF-1 level and a particular pattern of fat redistribution [45–47]. IGF-1 serves an important regulatory role in bone acquisition and maintenance of the adult skeleton, although its role in mesenchymal stem cell allocation towards the osteoblastic and adipocytic lineages remains unclear [46, 48]. Recent advances in genetic techniques to manipulate the mouse genome have resulted in several murine models that provide insights into the skeletal actions of IGF-1 and its potential interaction with other bone regulatory mechanisms.

One such animal model reflecting the relationship of IGF-1 with bone and fat consists of the congenic B6.C3H-6T (6T) mouse, which is a C57BL/6J (B6) mouse that carries a region of the C3H/HeJ (C3H) sixth chromosome [49]. Compared to B6, the 6T strain is characterized by low BMD, increased marrow fat, a reduced serum IGF-1 concentration, and reduced mRNA levels of IGF-1. Interestingly, the PPARγ gene is within the carried-over C3H-like region. Moreover, our recent results suggest that IGF-1 production in bone is under the control of the PPARγ gene [50].

## ROLE OF MARROW FAT AND ITS SIGNIFICANCE FOR THE MARROW MICROENVIRONMENT

As mentioned above, the PPAR-γ transcription factor is essential for both extramedullary and bone marrow fat development [19, 25], yet bone marrow adipocyte biology and function are not well understood. The marrow adipocyte phenotype is similar to that of adipocytes present in white and brown fat tissues, but the unique location of these cells in bone directs their more specialized functions [3]. For years, marrow fat was merely considered as a cellular component of bone that served a passive role by occupying a space no longer needed for hematopoiesis. However, recent developments suggesting that marrow fat plays an essential role as an endocrine organ involved in lipid and glucose metabolism place marrow fat under a new research spotlight. With advancing age, fat infiltrates bone marrow cavities, especially in the long bones [51]. From the perspective of adipokine production and glucose utilization, which is similar to white and brown fat, it is likely that marrow fat serves a variety of endocrine functions.

A relatively well-characterized role of marrow adipocytes is to support hematopoiesis by producing the necessary cytokines and providing heat for hematopoietic cell development. In addition, marrow fat may participate in lipid metabolism by clearing and storing circulating triglycerides and may provide a localized energy reservoir for emergency situations affecting, for example, osteogenesis (eg, bone fracture healing) [3]. Marrow adipocytes also produce several cytokines, but two adipokines, whose expression is under the PPAR-γ control, leptin and adiponectin, are currently the focus of increased attention as possible regulators of bone mass.

Leptin is produced by fat cells, and its primary role is the regulation of satiety through the effects on central nervous system [52]. Leptin expression increases during a starvation period resulting in decreases in growth, fertility, and bone mass; its expression decreases when energy intake increases. Leptin is thought to regulate bone mass through two alternative pathways: one involving a direct stimulatory effect on bone growth, when acting on bone cells through its receptors; and another, which is indirect, involving a hypothalamic relay that suppresses bone formation, when acting on central nervous system [52]. Thus, when acting locally on bone, leptin increases BMD, bone mineral content (BMC), and bone-formation rate, while it decreases the number and the size of bone marrow adipocytes [52]. In contrast, when injected into a hypothalamic ventricle, leptin decreases bone mass in the spine [53]. This activity is presumably mediated via β2-adrenergic receptors signaling, which regulates the expression of RANKL in osteoblasts [54].

Another adipokine, adiponectin, was recently discovered to be an insulin-sensitizing hormone produced by fat tissue [55]. Clinical studies implicate adiponectin as an independent predictor of bone mass; circulating levels of adiponectin correlate inversely with bone mass in humans [56]. Adiponectin and its receptors, similar to leptin and its receptors, are expressed by cells of the osteoblast lineage [57–60]. In vitro, adiponectin inhibits adipocyte formation and stimulates osteoblast proliferation and differentiation via the MAPK signaling pathways [59], however adiponectin-deficient or transgenic for its expression mice did not show bone abnormalities [60]. Since adiponectin can act on bone through either an autocrine/paracrine pathway and/or an endocrine pathway as a hormone secreted from fat tissue, Shinoda et al. concluded that adiponectin may have three distinct actions on bone: a positive action of locally produced adiponectin through an autocrine/paracrine pathway, a direct negative effect of circulating adiponectin, and a positive indirect action of circulating adiponectin via the enhancement of insulin signaling [60].

## EVIDENCE FOR THE RECIPROCAL RELATIONSHIP BETWEEN BONE LOSS AND OSTEOBLAST AND ADIPOCYTE DEVELOPMENT

Accumulating in vivo and in vitro evidences support the hypothesis that increased adipocyte formation occurs at the expense of osteoblast development. In humans, the association between bone loss and increased marrow adiposity is visible not only during aging, but also during conditions of skeletal disuse, such as microgravity or paraplegia [51, 61, 62]. In animals, skeletal unloading results in bone loss, which is also associated with an increase in the marrow fat compartment [63–66].

In contrast, the lack of adipose tissue has been associated with increased bone formation. In patients with congenital generalized lipodystrophy, a lack of body fat is accompanied by skeletal abnormalities, such as increased bone density, a thickened calvarium, and scoliosis [67, 68]. An animal model of lipodystrophy due to a hypomorphic mutation in the PPAR-γ gene exhibits both decreased marrow fat content and increased bone mass [26]. On the other hand, embryonic fibroblasts carrying a null mutation in the PPAR-γ gene spontaneously differentiate towards osteoblasts and do not possess the capability to differentiate towards adipocytes [25]. Strong evidence for a reciprocal relationship between adipocyte formation and bone loss is provided by studies that have examined the effect of TZDs, highly specific PPAR-γ agonists, on bone and bone marrow cell differentiation, as described above [27–30]. In support of this evidence, we have previously demonstrated in an in vitro model of marrow mesenchymal cell differentiation (U-33/γ2 cells) that activation of the PPAR-γ2 isoform by rosiglitazone converted cells of the osteoblast lineage to terminally differentiated adipocytes and irreversibly suppressed both the osteoblast phenotype and osteoblast-specific gene expression [9].

In the SAMP6 mouse model of involutional osteopenia associated with early senescence, low bone mass results from a diminished ability of MSCs to differentiate towards osteoblasts [69, 70]. Simultaneously, MSCs of SAMP6 mice exhibit an increased commitment towards the adipocyte lineage [71]. The impaired marrow osteogenesis is associated with a reduction in endochondral, but not periosteal, new bone formation, which suggests a defective differentiation of osteogenic progenitors present in the bone marrow [72]. Importantly, this defect is completely corrected when bone marrow derived from normal nonosteopenic mice is transplanted into irradiated SAMP6 mice [73]. Allogeneic bone marrow transplantation resulted in histologically normal trabecular bone and bone density and restored circulating levels of interleukin (IL)-11, RANKL, and IL-6, all cytokines involved in the regulation of bone remodeling.

The terminal differentiation of MSC towards osteoblasts and adipocytes results from the selective activation of specific programs of gene expression, which are controlled by phenotype-specific transcription factors, such as Runx2 and PPAR-γ, respectively. However, the control of expression and the activity of these factors, and their precise role in MSC lineage allocation, remain poorly understood. The recent identification of TAZ (transcriptional coactivator with PDZ-binding motif) provides some insight into how the activity of transcriptional regulators may be controlled and suggests that TAZ may act as a molecular switch in the differentiation of MSC to osteoblasts and adipocytes [74, 75]. TAZ protein functions in the convergance of extracellular signals from the cytoplasm to the nucleus [74], where it binds to the large number of transcription factors including Runx2 and PPAR-γ [76]. Binding of TAZ to Runx2 strongly coactivates Runx2-dependent gene transcription, while binding to PPAR-γ suppresses PPAR-γ-dependent gene transcription. Interestingly, closely related to TAZ protein, Yes-associated protein, YAP, acts as a strong repressor of Runx2 transcriptional activity and osteoblast differentiation in a manner that requires Src/Yes kinases activity [77]. However, its effect on adipocyte differentiation and PPAR-γ activity remains to be determined. Nevertheless, TAZ and YAP transcriptional modulators are suggested to be functionally related to β-catenin with respect to their role in integration of extracellular, membrane, and cytoskeletal-derived signals to influence mesenchymal stem cell fate [74].

Recent discoveries identifying an important role for the Wnt signaling pathway in postnatal bone accrual, by regulating osteoblast and osteoclast development, have provided major advances in our understanding of skeletal biology [78, 79]. Wnts are soluble glycoproteins that engage receptor complexes composed of Lrp5/6 and frizzled proteins, which induce a cascade of intracellular events that stabilize β-catenin, facilitating its transport to nuclei where it binds Lef1/Tcf transcription factors, and alters gene expression to promote osteoblast expansion and function. The first indication that Wnt signaling plays a critical role in bone formation came from human studies where inactivating mutations in the Wnt coreceptor LRP5 were shown to cause osteoporosis [80]. In contrast, gain of function mutations in LRP5 that increase Wnt signaling results in higher bone density in humans and mice [81, 82]. The Wnt pathway has also been implicated in the regulation of lineage allocation of MSC. Animals that express Wnt10b under the control of FABP4 in marrow are characterized by high bone mass, which is maintained during aging [83, 84]. Interestingly, Wnt 10b suppresses PPAR-γ expression and adipocyte development [83] and vice versa, PPAR-γ2 suppresses Wnt10b expression in U-33/γ2 cells [4]. Recent findings indicate that Wnt pathway not only regulates osteoblast development towards bone-forming cells, but it also controls osteoblast support of osteoclastogenesis [85, 86].

## EVIDENCE FOR NONRECIPROCAL BONE LOSS AND OSTEOBLAST AND ADIPOCYTE DIFFERENTIATIONS

In some circumstances, osteoblast and adipocyte differentiations may have a nonreciprocal nature. Recently, we demonstrated that administration of the selective TZD netoglitazone to animals resulted in extensive accumulation of marrow fat, but did not affect bone mass [5]. Similar findings were reported previously by Tornvig et al [87], who demonstrated that the administration of another TZD, troglitazone, to apolipoprotein E-deficient mice for 10 months did not affect bone mass, although it increased the number of marrow adipocytes and appeared to affect the marrow hematopoietic compartment. These data suggest that in vivo antiosteoblastic and proadipocytic activities of PPAR-γ can be independently activated by selective PPAR-γ modulators.

The nonreciprocal character of osteoblast and adipocyte differentiations is also supported by several animal models of bone mass regulation that are not directly related to PPAR-γ activity in MSCs. Mice deficient in 11β-hydroxysteroid dehydrogenase type 1 (HSD1^−/−^), an enzyme that converts inactive cortisone into active cortisol, exhibit normal bone formation and bone loss with aging in the absence of marrow adipocytes [88]. Conversely, overexpression of the transcriptional regulator δFosB in cells of the osteoblast lineage resulted in an increased number of osteoblasts and increased bone formation, with no effect on the number of marrow adipocytes [89]. In another murine model, deletion of the early B-cell factor gene, *EBF1*, results in a significant increase in osteoblast number and bone formation, in the face of the marrow cavity being filled with fat [90]. In total, these data suggest that the Janus-like osteoblast-adipocyte relationship is more complex than first thought and likely subject to selective regulation.

## DIVERGENT EFFECT OF PPAR-γ ACTIVATORS ON THE PROADIPOCYTIC AND ANTIOSTEOBLASTIC ACIVITIES

The ligand-binding pocket of PPAR-γ is promiscuous and binds a variety of molecules with different affinities [24]. We showed that PPAR-γ2 activation in osteoblast cells using natural and artificial ligands with distinct pharmacophores and binding affinities resulted in a divergent activation of the proadipocytic and antiosteoblastic activity of PPAR-γ2 [4]. For example, using a variety of oxidized linoleic acid derivatives (eg, its epoxy-, hydroxy- and dihydroxy-derivatives) we were able to demonstrate that the proadipocytic and antiosteoblastic activities of PPAR-γ2 can be separated. These results suggested that PPAR-γ2 effects on osteoblast and adipocyte phenotypes are mediated by distinct regulatory pathways that are differentially modulated depending on the nature of the ligand. Moreover, they suggested that there may be selective PPAR-γ2 modulators that have beneficial activities as insulin sensitizers, without adverse effects on bone. Therefore, we have tested whether any of the available FDA-approved antidiabetic TZDs also modulate PPAR-γ2 activities differently.

**Figure 2:**
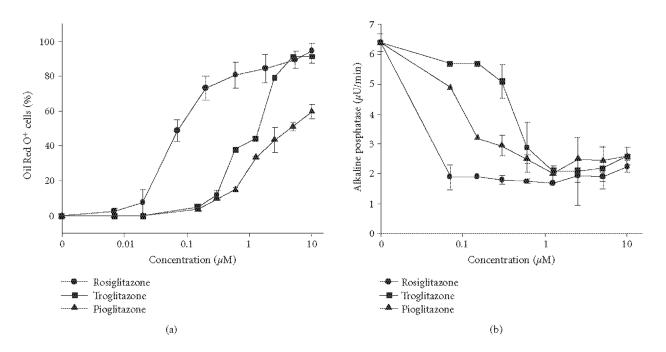
The effect of tested glitazones on adipocyte (a) and osteoblast (b) phenotypes of U-33/γ2 cells. U-33/γ2 cells represent marrow mesenchymal bipotential progenitor cells, which differentiation towards osteoblast and adipocyte is under the control of PPAR-γ2 transcription factor. Cells were treated for 3 days with different doses of tested PPAR-γ agonists and cultures were either stained for fat with Oil Red-O or subjected to alkaline phosphatase enzyme activity assay as previously described [4].

Using U-33/γ2 cells, in which osteoblast and adipocyte differentiation is under the control of constitutively expressed PPAR-γ2 [4, 9], we compared the antiosteoblastic and proadipocytic activities of troglitazone, pioglitazone, and rosiglitazone. The proadipocytic activity was measured as number of U-33/γ2 cells accumulating fat, and antiosteoblastic activity was measured as the suppression of alkaline phosphatase enzyme activity, in response to treatment with different doses of tested TZD. As showed in Figure 2, U-33/γ2 cells responded to this treatment in a dose-dependent manner and the antiosteoblastic and proadipocytic activities of tested TZDs correlated with their ligand binding affinity for PPAR-γ (rosiglitazone (EC_50_ = 0.04 μM) > pioglitazone (EC_50_ = 0.5 μM) > troglitazone (EC_50_ = 0.8 μM)) [24], with the exception to troglitazone, which appeared to have higher proadipocytic activity than pioglitazone.

Next, we measured the effect of TZDs on the expression of adipocyte and osteoblast signature genes using quantitative real-time PCR. We tested their effect on gene expression in U-33/γ2 cells and primary bone marrow cultures in concentrations that induced fat accumulation in 50% of U-33/γ2 cells. As shown in Table 1, the effects of tested TZDs, at doses which were equally effective for fat accumulation in U-33/γ2 cells, were similar. Although primary bone marrow cells responded to these treatments with a different magnitude than U-33/γ2 cells, all tested TZDs equally induced both proadipocytic and antiosteoblastic properties of PPAR-γ in both U-33/γ2 and primary bone marrow cells.

These effects are in contrast to the effects of another TZD, netoglitazone [5]. Netoglitazone appears to be a synthetic PPAR-γ ligand that separates the proadipocytic and antidiabetic activities from the antibone activity in vivo. Netoglitazone administered at a dose equally effective as rosiglitazone in lowering blood glucose in a murine model of type 2 diabetes did not induce bone loss, affect changes in bone microarchitecture, or alter bone-specific gene expression. Interestingly, netoglitazone, which possesses weak proadipocytic activities in vitro effectively induced marrow adipocyte formation in vivo. Regardless of the discrepancies between the in vitro and in vivo proadipocytic effects of netoglitazone, these results indicate that it is possible to separate the proadipocytic and antiosteoblastic activities of PPAR-γ in vivo. They also suggest that in vivo, at least some of the marrow cells are responsive to netoglitazone and thereby mediating the proadipocytic activity. Interestingly, it appears that this population of cells is not involved in production of bone-forming osteoblasts. Collectively, these data suggest that these effects are modulated by the cellular environment and/or the availability of specific cofactors required for PPAR-γ activity [91].

## CONCLUSIONS

Osteoporosis, obesity, and diabetes are the most common pathologies seen in highly industrialized countries and the cost impact to treat these diseases is enormous and still growing. Since PPAR-γ is positioned at the cross-roads of the control of bone mass, energy expenditure, and glucose metabolism, changes in its activity, which occur either naturally during aging or during antidiabetic therapy using TZDs, may result in unwanted effects on the skeleton. The attractive possibility to separate specific PPAR-γ activities may allow for the development of selective antidiabetic modulators that will also be safe for the skeleton. Such a possibility ensures that there will be a continued discovery effort to identify pharmacophores that will be of benefit for both bone and glucose metabolism.

## Figures and Tables

**Table 1:** The effects of TZDs on osteoblast and adipocyte gene markers.

Treatment	Cell type	PPAR-γ2	FABP4	Dlx5	Runx2	OC	Coll

Rosiglitazone^(a)^	U-33/γ2	4.0^(d)^	2, 558.0	0.18	0.23	0.01	0.26
	Bone marrow	74.8	94.4	0.27	0.14	0.13	0.18

Pioglitazone^(b)^	U-33/γ2	2.4	1, 857.0	0.15	0.21	0.01	0.19
	Bone marrow	367.8	84.0	0.40	0.39	0.16	0.14

Troglitazone^(c)^	U-33/γ2	2.9	2, 234.0	0.14	0.19	0.01	0.18
	Bone marrow	160.8	108.0	0.39	0.32	0.07	0.17

TZDs concentrations: ^(a)^1 μM; ^(b)^6 μM; ^(c)^10 μM; ^(d)^ values represent fold of gene expression in cells treated with TZDs versus untreated control.
